# High-efficiency nonlocal reflection-type vortex beam generation based on bound states in the continuum

**DOI:** 10.1093/nsr/nwac234

**Published:** 2022-10-22

**Authors:** Tongyu Li, Jiajun Wang, Wenjie Zhang, Xinhao Wang, Wenzhe Liu, Lei Shi, Jian Zi

**Affiliations:** State Key Laboratory of Surface Physics, Key Laboratory of Micro- and Nano-Photonic Structures (Ministry of Education) and Department of Physics, Fudan University, Shanghai 200433, China; State Key Laboratory of Surface Physics, Key Laboratory of Micro- and Nano-Photonic Structures (Ministry of Education) and Department of Physics, Fudan University, Shanghai 200433, China; State Key Laboratory of Surface Physics, Key Laboratory of Micro- and Nano-Photonic Structures (Ministry of Education) and Department of Physics, Fudan University, Shanghai 200433, China; State Key Laboratory of Surface Physics, Key Laboratory of Micro- and Nano-Photonic Structures (Ministry of Education) and Department of Physics, Fudan University, Shanghai 200433, China; Department of Physics, The Hong Kong University of Science and Technology, Hong Kong, China; State Key Laboratory of Surface Physics, Key Laboratory of Micro- and Nano-Photonic Structures (Ministry of Education) and Department of Physics, Fudan University, Shanghai 200433, China; State Key Laboratory of Surface Physics, Key Laboratory of Micro- and Nano-Photonic Structures (Ministry of Education) and Department of Physics, Fudan University, Shanghai 200433, China

**Keywords:** photonic crystal slab, vortex beam, bound states in the continuum

## Abstract

Momentum-space polarization vortices centered at symmetry-protected bound states in the continuum of a periodic structure, e.g. photonic crystal slab, provide a novel nonlocal approach to generate vortex beams. This approach enjoys a great convenience of no precise alignment requirements, although the generation efficiency of the nonlocal generators requires further optimization before the practical application. In this work, we propose a temporal-coupled-mode-theory-based guideline for high-efficiency nonlocal reflection-type vortex generator design. The conversion efficiency of the vortex beam is found to be limited by the ratio of the radiative loss to the intrinsic absorption in practical systems. To increase this ratio through mode selection and structure design, the photonic crystal slabs are theoretically designed and experimentally characterized, showing a maximum on-resonance conversion efficiency of up to 86%. Combining high efficiency with simple fabrication and no requirement for precise alignment, reflection-type photonic crystal slabs could offer a new and competitive way to generate vortex beams flexibly.

## INTRODUCTION

The orbital angular momentum (OAM) of light is a new degree of freedom that can be used to modulate propagating lights [1,2]. The vortex beam (VB), as one of the carriers of OAM, features a spiral wave front and a central zero-intensity singularity [3,4]. Applications of VBs have been extensively investigated in imaging [5], optical manipulation [6–8] and optical communication [9] since VBs were first discovered in 1992 [10]. To implement these applications, VB generation has been realized over a wide wavelength range [11–17], with VBs mostly being generated using structures with an alignment center.

These conventional methods follow a similar intrinsic motivation to generate VBs, which involves the manipulation of incident beams using observable spiral or rotating structures in real space to form the spiral phase distributions. Among these methods, the most direct approach is to use a spiral phase plate that has a radially varying thickness to accumulate the spiral propagation phase for normally incident plane waves and was first realized at millimetre-wave frequency [18]. As a feasible method for use in the visible light range, the q-plate is an inhomogeneous half-wave plate made from liquid crystal that has a rotating in-plane optical axis orientation; the q-plate will add an additional spiral phase distribution to the normal incident beam, while also performing its polarization transformation function [19]. A programmable version has been derived based on the same generating principle in the form of a spatial light modulator [20–22]. Additionally, spiral wave fronts can also be recovered using fork-grating-shaped interference patterns recorded by holograms [23]. In recent years, a more compact and integrable method, called the metasurface [24], has been demonstrated to be capable of performing VB generation in the near infrared and visible light ranges using individually designed and fabricated units. However, all the generators mentioned above require the beam centers to be carefully aligned with their respective geometric centers. In the visible region, the resulting alignment difficulties become significant in practical applications.

Recently, a new nonlocal VB generation method based on the use of momentum-space polarization vortices centered at the symmetry-protected bound states in the continuum [25–29] of periodic structures was proposed [30]. These periodic structures, e.g. two-dimensional photonic crystal (PhC) slabs, can reduce difficulties caused by beam alignment and high-precision fabrication requirements in practical applications, although the low generation efficiency that occurs in conventional methods also occurs in this method.

## RESULTS AND DISCUSSION

To improve VB generation efficiency, by intricately designing individual units at micro- or nanometer scale, metasurface generators have realized great progress [31,32]. Similar to the metasurface, the perfect mirror can also be introduced to block the transmission channels in nonlocal VB generation systems, which will then only transfer the incident energy to the reflection channel. We theoretically proposed that VB generation using single resonances and a perfect mirror in a reflection-type system can achieve $100\%$ cross-polarized conversion efficiency [33]. However, when the practical applications at visible and near-infrared wavelengths are considered, the absorption of the metal mirror will inevitably cause a loss that will reduce the conversion efficiency of such a VB generator greatly. In this work, we propose a general picture based on temporal coupled mode theory (TCMT) [34] to improve the VB generation efficiency of reflection-type generators with intrinsic absorption, which is determined by the ratio of the radiative loss to the intrinsic absorption. Based on this picture, mode selection and structure design are employed to increase this ratio efficiently. In both simulations and experiments, the maximum on-resonance conversion efficiency of the designed reflection-type PhC VB generators reaches up to $86\%$. The VB profiles generated at different wavelengths and in different working regions are also observed experimentally.

To illustrate the mechanism of a nonlocal VB generator, considering a PhC slab for a particular frequency, a resonant mode, e.g. a guided resonance [35], above the light cone and below the diffraction limit will radiate into free space whose state of polarization (SOP) can therefore be defined in the far field. Close to the at-Γ bound state in the continuum (BIC), the SOPs of these guided resonances are nearly linearly polarized [36]. The major axes of these states rotate around the BIC singularity, driven by the rotational symmetry of the PhC slabs [30], forming strong polarization anisotropy in paraxial fields that leads to an extrinsic spin-orbit interaction [37–40]. When circularly polarized light impinges upon a PhC slab and interacts with these guided resonances around the BIC, the converted cross-polarized light will then gain Pancharatnam–Berry phases [41] and form a spiral wave front. The topological charge *l* of the spiral wave front is thus determined by the polarization charge *q* of the BIC, where *l* = −2 × *q* [30]. In addition, the polarization charge *q* of a BIC has been studied and has been shown to depend on the real space symmetry of the PhC slabs and the symmetry representation of the BIC mode [26,27].

For this generation principle, the VB generation efficiency is limited by the conversion efficiency of the resonance process. The topological charge *l* of the spiral wave front is thus determined by the polarization charge *q* of the BIC, where *l* = −2 × *q* [30]. In addition, the polarization charge *q* of a BIC has been studied and has been shown to depend on the real space symmetry of the PhC slabs and the symmetry representation of the BIC mode [26,27].

In TCMT, the dynamics of a resonance $A(\boldsymbol{k}_{||})$ on an isolated band [34] in a system with material absorption can be formulated as


(1) \begin{eqnarray*} \frac{\mathrm{d} A }{\mathrm{d}t}=\left(-i\omega _0-\gamma _0-\sum _{i=s\!,\!p}\gamma _i\right) A +\boldsymbol{K}^T |s_+ \rangle , \end{eqnarray*}

(2) \begin{eqnarray*} |s_- \rangle =\boldsymbol{C} |s_+ \rangle +\boldsymbol{D} A =\boldsymbol{S} |s_+ \rangle . \end{eqnarray*}

Here, *A* is the amplitude of the resonance with eigenfrequency ω_0_,γ_0_ is the intrinsic loss due to material absorption and γ_*i*_ is the radiative loss. The column vectors |*s*_+_〉 = (*s*_+_, *p*_+_)^*T*^ and |*s*_−_〉 = (*s*_−_, *p*_−_)^*T*^ refer to the amplitudes of the incident and reflected light, respectively. We select the basis of *s* and *p* polarization to describe the system’s two orthogonal energy ports to free space. The two-by-two matrix *C* provides the generalized background scattering matrix, with the two-by-two matrix $\boldsymbol{S}$ as the generalized scattering matrix. For a single resonance above the light cone and below the diffraction limit, the coupling matrices $\boldsymbol{K}=(k_s,k_p)^T$ and $\boldsymbol{D}=(d_s,d_p)^T$ are both two-by-one matrices. We can then certainly use the orthogonal coupling coefficients (*d_s_*, *d_p_*) to describe the SOP of the resonance[30].

Considering the energy conservation and time-reversal consideration, there are several constraints for the parameters above, which are written as


(3) \begin{eqnarray*} \boldsymbol{D}^\dagger \boldsymbol{D}=2\gamma _i, \end{eqnarray*}

(4) \begin{eqnarray*} \boldsymbol{D} = \boldsymbol{K}, \end{eqnarray*}

(5) \begin{eqnarray*} \boldsymbol{C}\boldsymbol{D}^*=-\boldsymbol{D}. \end{eqnarray*}

Because of the fact that the SOPs of the resonances near the at-Γ bound states in the continuum are nearly linearly polarized [36], we then assume that the discussed resonance only coupled to the *s*-polarized port: γ_*i*_ = $\gamma_s$. Then, we obtain the results from the scattering matrix:


(6) \begin{eqnarray*} |d_s| = \sqrt{2\gamma _s},\qquad |d_p|= 0, \end{eqnarray*}

(7) \begin{eqnarray*} {\bf {S}}&=& \Bigg( \begin{array}{cc} 1-{2\gamma _s}/{[-i(\omega -\omega _0)+\gamma _0+\gamma _s]}&0\\ 0&1 \end{array}\Bigg)\\ &&\quad\quad\quad e^{i\phi } = \Big(\begin{array}{cc} s_{11}&0\\ 0&1 \end{array}\Big) e^{i\phi}. \end{eqnarray*}

We consider the resonances along the *k_y_*-axis direction under near normal incidence to discuss their responses with different polarized incidences. In this case, the *x* (*y*) polarizations are nearly equivalent to the *s* (*p*) polarizations, with *d_x_* ≈ *d_s_*, *d_y_* ≈ *d_p_* = 0. The reflectance and absorptance that occur under *x*- (*s*-) and *y*- (*p*-)polarized incidence conditions are


(8) \begin{eqnarray*} R_s(\omega )=|s_{11}|^2 , \end{eqnarray*}

(9) \begin{eqnarray*} A_s(\omega )=1-R_s(\omega )=\frac{4\gamma _0\gamma _s}{(\omega -\omega _0)^2+(\gamma _0+\gamma _s)^2}.\!\!\!\!\!\! \\ \end{eqnarray*}

Therefore, TCMT parameters ($\gamma_s$, γ_0_ and ω_0_) can then be obtained from reflectance spectra.

For a polarization vortex near the at-Γ BIC, the major axes of the SOPs would rotate around the Γ point. Considering a ψ angle between the major axes of the SOP and the *x* axes, similar conclusions are obtained via a rotational transformation of the axis. By transforming the basis from the *s* (*p*) basis |*s*〉_*s, p*_ to the helical basis |*s*〉_*l, r*_ [30], we can then describe the corresponding cases under circularly polarized incidence conditions:


(10) \begin{eqnarray*} |s_- \rangle _{l,r} &=& \boldsymbol{T}^{-1}_{helical}\boldsymbol{R}^{-1}(\psi )\boldsymbol{S}\boldsymbol{T}_{helical} \boldsymbol{R}(\psi ) |s_+ \rangle _{l,r} \\ &=&\frac{1}{2}\! \Bigg(\!\! \begin{array}{ll}s_{11}+1 & (s_{11}-1)e^{i2\psi }\\ (s_{11}-1)e^{-i2\psi } & s_{11}+1 \end{array}\!\!\Bigg)\! |s_+ \rangle _{l,r}.\\ \end{eqnarray*}

If the incidence is circularly polarized, it can be found that the cross-polarized terms would gain a geometric phase [41] factor with different values of ψ, and the corresponding cross-polarized conversion efficiency is


(11) \begin{eqnarray*} R_c(\omega ) = \bigg | \frac{s_{11}-1}{2}\bigg | ^2=\frac{\gamma _s^2}{(\omega -\omega _0)^2+(\gamma _0+\gamma _s)^2}.\!\!\!\!\!\! \\ \end{eqnarray*}

**Figure 1.**
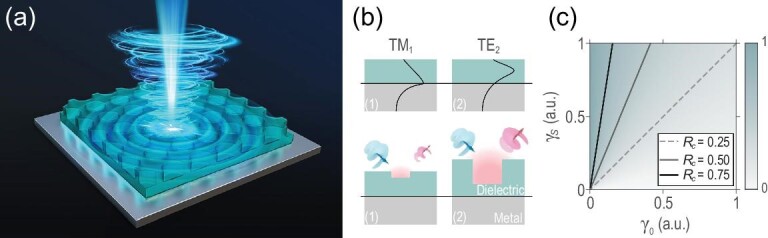
(a) Schematic view of the reflection-type PhC-VB generator. (b) In the top panel we plot the schematic electric field distributions of the TM_1_ band and TE_2_ band in the air-dielectric-metal structure. The field of TM_1_ gathers on the surface of the metal mirror, causing large intrinsic absorption, i.e. $\gamma _0^{(1)}>\gamma _0^{(2)}$. Bottom panel: the large scattering rate caused by the geometric structure in a unit cell corresponds to large radiative loss, i.e. $\gamma _s^{(1)}<\gamma _s^{(2)}$. (c) Cross-polarized conversion efficiency *R_c_* map calculated using (11) with normalized $\gamma_0$ and $\gamma_s$. Here *R_c_* reaches high efficiency when $\gamma_s$$\gg$$\gamma_0$.

It is clearly shown here that the cross-polarized conversion efficiency will approach $100\%$ on resonance when $\gamma_0$ vanishes. Practically, a reflection-type generator will still achieve a high conversion efficiency easily when $\gamma_s$$\gg$$\gamma_0$, as shown in the top left corner of Fig. 1(c). Because $\gamma_0$ relates to the intrinsic loss of the system and $\gamma_s$ to the scattering of the guided resonance, mode selection and structure design are proposed to inhibit $\gamma_0$ and enlarge $\gamma_s$. For instance, as plotted in Fig. 1(b), the electric field of the first transverse-magnetic (TM)-like band is located on the mirror surface (see the online supplementary material), which leads to a large loss, while that of the second transverse-electric (TE)-like band approaches zero in the vicinity of the mirror, which leads to a smaller $\gamma_0$. With regard to $\gamma_s$, the radiative loss of the guided resonance corresponds to the light scattering caused by the periodic structures, for which the designed geometric parameters of the unit cells require further refinement.

To verify the above guideline, we designed reflection-type two-dimensional PhC slabs with the metallic mirror and characterized their conversion efficiencies both theoretically and experimentally. The studied reflection-type PhC slab consists of a flat silver mirror coated with an adhesion layer and a periodically etched Si_3_N_4_ dielectric layer. The silver mirror is formed by a 300-nm-thick film that was evaporated on a silicon substrate, above which a 25-nm-thick SiO_2_ film was coated as an adhesion layer. The periodic structures are circular air hole arrays arranged in a square lattice with their fixed period of 460 nm; these structures were fabricated by electron beam lithography on dielectric layers.

**Figure 2.**
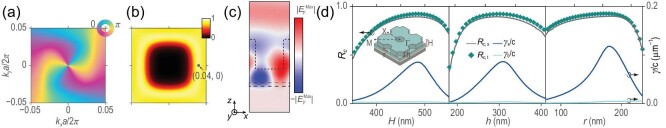
(a and b) The iso-frequency contour of phase and cross-polarized conversion efficiency at a wavelength of 797 nm under circular incidence (*h* = 330 nm, *r* = 160 nm and *H* = 500 nm). (c) Side view of the *E_y_* distribution of band TE_2_ at $\boldsymbol{k}_{||}a/2\pi =(0.04,0)$. The dashed lines outline the PhC slab cross section in one unit cell. (d) The variation of *R_c_* at $\boldsymbol{k}_{||}a/2\pi =(0.04,0)$ on band TE_2_ with different structure parameters. Gray lines represent simulated cross-polarized conversion efficiency, light blue lines and dark blue lines represent $\gamma_s$ and $\gamma_0$ obtained from TCMT, and green squares represent calculated cross-polarized conversion efficiency from (11) using $\gamma_s$ and $\gamma_0$. The schematic diagram of the PhC slab structure is shown in the inset; green part is an etched Si_3_N_4_ layer, yellow part is a SiO_2_ film and gray part is a silver mirror.

To inhibit the $\gamma_0$ of the system, the TE_2_ band was selected for the VB generation. As a VB generator, the momentum-space polarization field of the selected TE_2_ band is shown in the online supplementary material, with the BIC being centered at the Γ point and the charge of the polarization vortex being +1. Considering the circularly polarized incidence, the cross-polarized light reflected via such a polarization vortex will gain a 4π spiral phase along any clockwise loop around the BIC singularity. As examples, the calculated phase distribution and the cross-polarized conversion efficiency iso-frequency contour of the TE_2_ band were plotted in Fig. 2(a) and (b), respectively. To demonstrate the inhibition of $\gamma_0$, the electric field of the TE_2_ band at $\boldsymbol{k}_{||}a/2\pi =(0.04,0)$ is shown in Fig. 2(c). The electric field was demonstrated to gather sparingly above the silver film surface, indicating that only a small level of material absorption occurred in the system.

To enhance $\gamma_s$ further in the selected TE_2_ band, three geometrical parameters in the PhC slab required to be determined: the etched depth *h*, the hole radius *r* and the dielectric layer thickness *H*. We focused on the conversion efficiency at $\boldsymbol{k}_{||}a/2\pi =(0.04,0)$ on the iso-frequency contour to determine the parameters that provided high conversion efficiency. Starting with the PhC slab with parameters *h* = 300 nm, *r* = 160 nm and *H* = 480 nm, each of the parameters were varied within certain ranges to observe the variation in *R_c_*. On the one hand, *R_c_* at $\boldsymbol{k}_{||}a/2\pi =(0.04,0)$ can be calculated with the different PhC parameters using rigorous coupled-wave analysis (RCWA) [42], as plotted with gray lines in Fig. 2(d). On the other hand, based on (11), the conversion efficiency was calculated using $\gamma_0$ and $\gamma_s$ that can be extracted from the reflectance spectra (see the online supplementary material). The obtained $\gamma_0$ and $\gamma_s$ values are depicted with light blue lines and the dark blue lines in Fig. 2(d), respectively. It was found that $\gamma_0$ is near zero, thus verifying the small level of absorption, and $\gamma_s$ was strongly influenced by the structure. Using the extracted $\gamma_0$ and $\gamma_s$, the *R_c_* spectra were then calculated using (11) and are indicated with green square markers in Fig. 2(d), which matched the simulated results well. With respect to the different selected parameters, *R_c_* varied from $50\%$ up to nearly $90\%$ and remained higher than $80\%$ over a wide parameter range, forming large flat steps in the middle of each panel. The high-efficiency flat step was also demonstrated experimentally and is shown in the online supplementary material.

**Figure 3.**
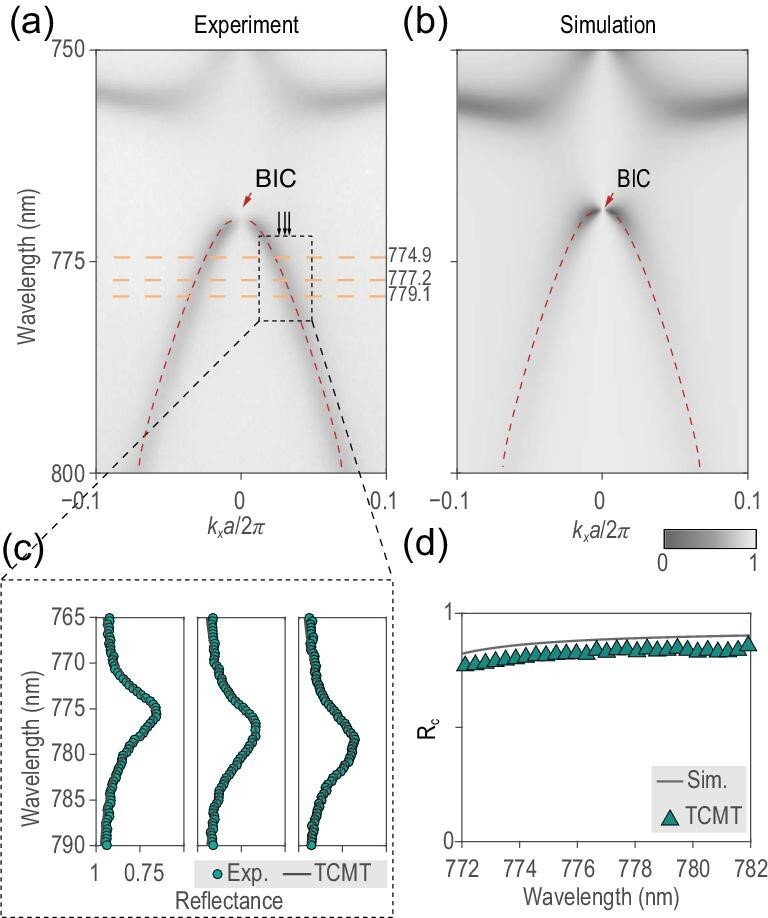
(a and b) The measured and simulated angle-resolved reflectance spectra along Γ-*X* under *s*-polarized incidence. Bands TE_2_ were marked out by dashed lines. The vanishing point of band TE_2_ corresponds to a central BIC. (c) Detailed experimental reflectance spectra at three different wave vectors, $\boldsymbol{k}_{||}a/2\pi =(0.027,0)$, (0.031,0) and (0.034,0) (green dots), and corresponding fitting curves (gray lines) with TCMT. (d) The *R_c_* obtained from measured band TE_2_ at different wavelengths (green squares) whose maximum on-resonance conversion efficiency is $86\%$, and the simulated *R_c_* spectra (gray line).

By selecting a group of structural parameters on this flat step, i.e. *h* = 337 nm, *r* = 186 nm and *H* = 519 nm, the PhC slab was fabricated and characterized experimentally. As indicated by the polarization maps plotted in the online supplementary material, the proposed TE_2_ band in the vicinity of the at-Γ BIC is only excited by *s*-polarized incident light along Γ-*X*, and the measured and calculated angle-resolved reflectance spectra under the *s*-polarized incidence along the Γ-*X* direction are illustrated in Fig. 3(a) and (b), respectively. The optical measurements were performed using our home-made angle-resolved imaging spectroscopy system [43–45], and the theoretical spectra were simulated via RCWA. The marked TE_2_ band was used to generate the VB. The central frequency ω_0_ and the corresponding $\gamma_s$ and $\gamma_0$ values were extracted via numerically fitting the reflectance spectra at different wave vectors; examples are shown in Fig. 3(c). The cross-polarized conversion efficiency *R_c_* in the experiments was obtained from the measured *s*-polarized reflectance spectra using (11). Obtained *R_c_* values of the experimental TE_2_ band at the different wavelengths are marked as green squares in Fig. 3(d), while the simulated *R_c_* spectra along Γ-*X* under circularly polarized incidence are plotted as gray lines, showing high conversion efficiency over the entire TE_2_ band and also showing good agreement with the theory; a detailed comparison is shown in the online supplementary material.

**Figure 4.**
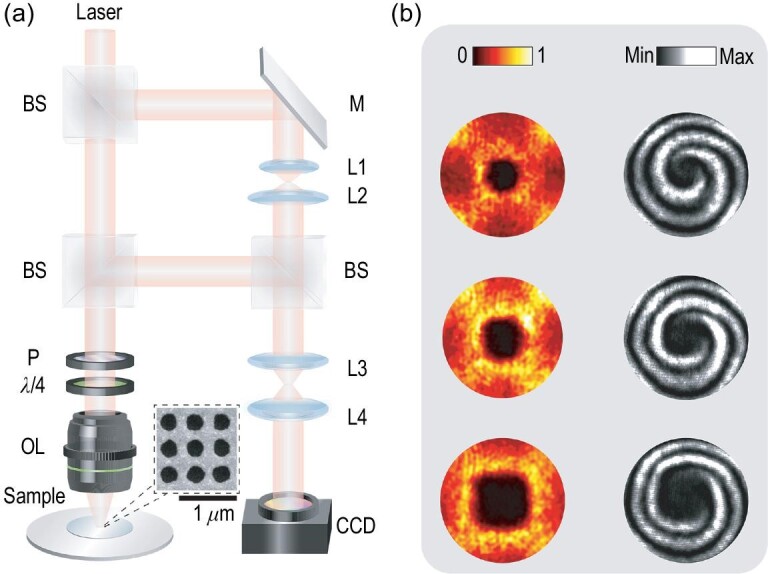
(a) The experimental setup. BS, beam splitter; L1–L4, lenses; M, mirror; OL, objective lens; P, polarizer; λ/4, 1/4 wave plate. Inset: the scanning electron microscope image of the PhC slab sample. (b) The measured results of fabricated PhC slabs. Left panel: the measured cross-polarized conversion efficiency. Right panel: the measured interference fringes between the generated spiral wave front and the reference wave front, where a singularity can be found in the center of the profile with 2 topological charge. From top to bottom, the wavelengths of incident LCP light were 774.9, 777.2 and 779.1 nm, respectively.

To further characterize the VB profiles, we built a home-made reflection-type Fourier-optics-based imaging system that had an imaging mode and an interferometer mode; a schematic diagram of this system is shown in Fig. 4(a). By taking the mirror in the reference path down, the system was firstly operated in imaging mode to measure the cross-polarized conversion efficiency iso-frequency contour, i.e. the beam profiles in the far field. In this mode, the reference light was not introduced into the measurement process. The incident laser was circularly polarized by a circular polarizer module (left-handed circularly polarized, or LCP) that included a linear polarizer and a 1/4 wave plate (1/4λ), and was then focused on our fabricated PhC slab through an objective lens (OL). The focused light impinged upon the PhC slab in all the directions allowed by the numerical aperture and was coupled with the *k*-variant guided resonances surrounding the BIC, causing the reflected light on these resonances to be strongly cross-polarized. Reversely passing through the same OL and circular polarizer module, the reflected light was then Fourier transformed into the momentum space. The beam profile was finally detected by a charge-coupled device (CCD) after imaging using a set of confocal lenses (L3 and L4). By varying the wavelength of the incident laser, beam profiles at 774.9, 777.2 and 779.1 nm, marked as orange dashed lines in Fig. 3(a), are plotted in the left panel in Fig. 4(b). It was observed that the generated beams had doughnut-shaped far-field profiles with central zero-intensity points, which is a key feature of VBs. Following the dispersion of the TE_2_ band, the beam profile extended gradually in the momentum space and maintained high conversion efficiency.

Then, we switched on the mirror slightly to apply reference light for the operating interferometer mode. The linearly polarized reference light was used to illuminate the CCD to obtain interference patterns between the generated spiral wave front and the reference wave front. The interference patterns measured at the three wavelengths above are illustrated in the corresponding right panel of Fig. 4(b), where two obvious spiral arms can be observed at the profile center. This combination of a doughnut-shaped beam profile and the existence of spiral arms verified that the cross-polarized beam reflected by our fabricated PhC slab was a VB with a topological charge *l* = −2. Performing a Laguerre–Gaussian modal decomposition of the experimentally generated VB beam at 774.9 nm indicated that the energy of the *l* = −2 components accounted for $81.6\%$ of the total energy of the generated beam, details of which are presented in the online supplementary material.

**Figure 5.**
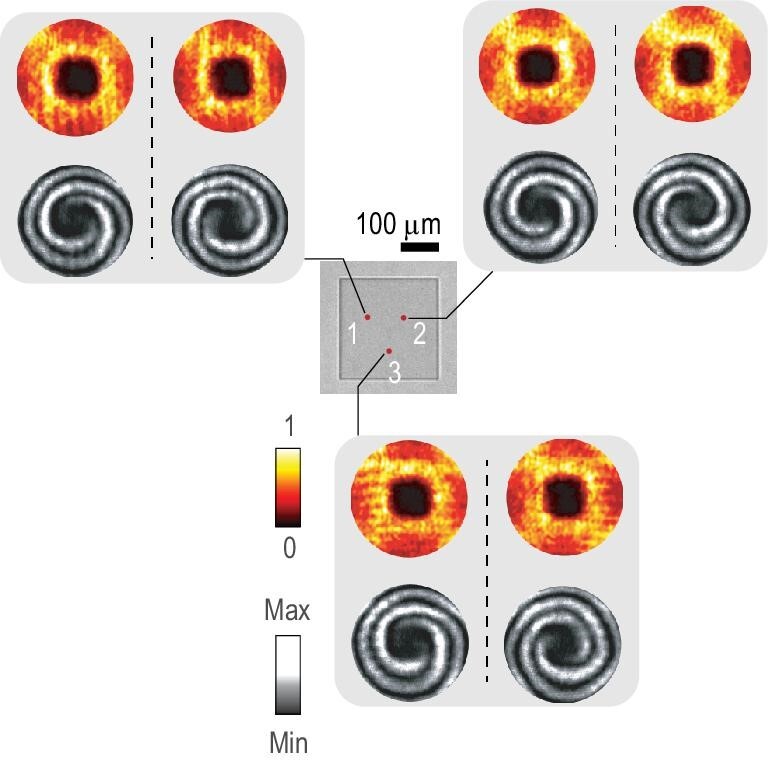
Inset: three measurement regions (red dots) on the optical image. The measured cross-polarized conversion efficiency and interference patterns in momentum space with LCP and RCP incidence respectively plotted in the left and right parts of the three gray panels.

Furthermore, selecting three different regions on our fabricated PhC slab to perform the measurements, the beam profiles and the interference patterns were measured repeatedly with the incident light at 774.9 nm. The measurement regions are highlighted with red dots in the optical image shown in Fig. 5. By rotating the 1/4 wave plate, we demonstrated beam generation with both right-handed circularly polarized (RCP) and LCP incidence. The beam profiles and the interference patterns remain unchanged, indicating that high-efficiency generation of the VB using a singularity in momentum space is nonlocal, i.e. it does not rely on the working position.

Here, we present some discussion. First, the nonlocal VB generation using our proposed principle is related to the at-Γ BIC, which can be realized flexibly at different wavelengths by tuning or even by simply scaling the structural parameters to make the band fall into the desired working region (see the online supplementary material). Additionally, we further make some comparison with some previous works that have made efforts to generate VBs via manipulations in momentum space. In these works, multiple-beam interference and spin-pseudospin coupling were used to selectively excite pseudospins located at K or K’ valleys in artificial photonic graphene to generate VBs [46,47]. Note that these detected angular momentums did not result from the nonzero Berry curvature at the K and K’ valleys, but instead resulted from the initial pseudospins of the Dirac system, while ours come from the polarization vortex existing around the at-Γ BIC. For practical applications, their methods may be suitable for generating VBs with oblique incidence, while our PhC-based methods are feasible with normal incidence.

## CONCLUSIONS

In conclusion, we have reported a TCMT-based approach for the design of high-efficiency nonlocal reflection-type PhC slabs for VB generation. In this approach, selection of a particular working mode of the PhC slab can efficiently reduce the inevitable absorption loss of the metal mirror in both the visible and near-infrared wavelength ranges, and further structure design will boost the conversion efficiency. Using this approach, VBs can be efficiently generated using PhC slabs with no alignment centers and a simple fabrication process, bringing VBs one step closer to practical application use, such as optical communication, imaging and quantum information processing.

## METHODS

Details are available in the online supplementary material.

## Supplementary Material

nwac234_Supplemental_File
