# Supplementary material for: High-efficiency nonlocal reflection-type vortex beam generation based on bound states in the continuum
Source: Natl Sci Rev. 2022 Oct 22;10(5):nwac234. doi: 10.1093/nsr/nwac234 (PMC10129391; doi:10.1093/nsr/nwac234)
Supplement: nwac234_Supplemental_File [file nwac234_supplemental_file.pdf]

Supplementary data for High-efficiency nonlocal reflection-type vortex beam generation based on bound states in the continuum

I. SUPPORTING EQUATIONS

A. Laguerre–Gaussian modal decomposition

Laguerre–Gaussian (LG) modes are one of eigenfunctions of paraxial wave equation, which consists of a complete orthogonal basis with circularly symmetric beam profiles. Phase fronts of these modes are spiral with the axis as the center which have been demonstrated to carry orbit angular momentums (OAMs) [1]. Thus, LG modal decomposition of arbitrary beam profile can be used to analysis its OAM compositions. By taking $+z$ as the propagating direction, x component of electric field of LG mode of order (l, p) can be expressed as $\mathbf{E}_{l,p}(r, \phi, z, t) = u_{l,p}(r, \phi, z)e^{-i(k_0 z - \omega t)}\hat{\mathbf{x}}$, where the scalar factor $u_{l,p}(r, \phi, z)$ is formulated as

$$u_{l,p}(r,\phi,z) = C_{l,p}\frac{1}{w(z)}\left(\frac{r\sqrt{2}}{w(z)}\right)^{|l|}\exp\left(-\frac{r^2}{w^2(z)}\right)L_p^{|l|}\left(\frac{2r^2}{w^2(z)}\right)\exp\left(-ik\frac{r^2}{2R(z)}\right)\exp(-il\phi)\exp(i\psi(z)), \tag{S1}$$

where $k = 2\pi n/\lambda$ is wave vector, $w(z) = w_0\sqrt{1 + (z/z_R)^2}$ is the beam radius with waist radius w_0 and Rayleigh range $z_R = \pi w_0^2 n/\lambda$, $R(z) = z[1 + (z_R/z)^2]$ is the radius of curvature, $L_p^{|l|}$ are generalized Laguerre polynomials, $\psi(z) = (2p + |l| + 1)\arctan(z/z_R)$ is Gouy phase and $C_{l,p} = \sqrt{2p!/[\pi(p + |l|)!]}$ is the normalized coefficient. Without loss of generality, we ignore the time dependent coefficient and set $z = 0$. Furthermore, Bra-Ket form $|u_{l,p}\rangle$ is used to represent LG mode of order (l, p).

LG modal decomposition for arbitrary field $|E(r, \phi)\rangle$ can be written as

$$|E(r,\phi)\rangle = \sum_{l,p} A_{l,p}|u_{l,p}\rangle, \tag{S2}$$

where expansion coefficients $A_{l,p}$ are obtained by the inner product $A_{l,p} = \langle u_{l,p},\, E(r, \phi)\rangle$. Different from plane wave decomposition, decomposition quality by using LG modes will be influenced by the choice for waist radius w_0. Some previous works have discussed how to choose a proper waist radius of LG modes and corresponding truncation order to obtain a stable recovered field[2, 3]. In the following decomposition, truncation order of p is chosen from 0 to 10, and l from -10 to 10. Some constants are scaled during decomposition: wavelength is scaled to 10^{-2}, waist radius of LG modes to 10^{-1} and truncation radius of input field to 0.43.

We first decomposed the simulated beam profile generated by PhC slab having the same structural parameters as that demonstrated in Fig. 4 and 5. We choose 774.9 nm as the working wavelength of PhC slab, as same as the middle row in Fig. 4. Original intensity and phase of the simulated beam profile are illustrated in the first row in Fig. S1(a) . Corresponding recomposed intensity and phase are illustrated in the second row. To visualize LG components of our generated beam, image of $|A_{l,p}|^2$ distribution is plotted in the bottom row. We summed up decomposition coefficients of LG modes carrying the same OAM to further illustrate OAM components of the generated beam, that is, $\sum_p |A_{l,p}|^2/\sum_{l,p}|A_{l,p}|^2$ is plotted as a histogram in the third row. It's clearly shown that $l = -2$ components account for the majority in the simulated beam profile, up to 95.8%, which means the proposed nonlocal vortex beam generation guideline could theoretically generate vortex beam with high purity of desired OAM as well as high conversion efficiency.

Decomposition is also performed in experimentally measured beam profile. Since decomposition with experimental data requires to the measured intensity data and phase data, here, we additionally perform the phase measurement. In experiment, off-axis digital hologram reconstruction method[4] is used to measure the phase distribution. In interference mode in Fig. 4(a), we rotated the mirror M to make the reference light obliquely shined on the CCD with an in-plane wavevector $\mathbf{k}_{||,ref.}$ to form clear and compact interference fringes. The intensity of the obtained interference pattern can be written as,

$$I_{int.} = I_{ref.} + I_{obj.} + \sqrt{I_{ref.}I_{obj.}}\{\exp\left[i(\phi_{obj.} + \mathbf{k}_{||,ref.}\cdot\mathbf{r})\right] + \exp\left[-i(\phi_{obj.} + \mathbf{k}_{||,ref.}\cdot\mathbf{r}))\right]\}. \tag{S3}$$

By Fourier transforming measured interference fringes digitally, we obtained one main peak and two satellite peaks in reciprocal space. By selecting one of satellite peaks, corresponding to $\exp\left[i(\phi_{obj.} + \mathbf{k}_{||,ref.}\cdot\mathbf{r})\right]$, and filtering out other regions, phase distribution of the object can be obtained by inverse Fourier transformation.

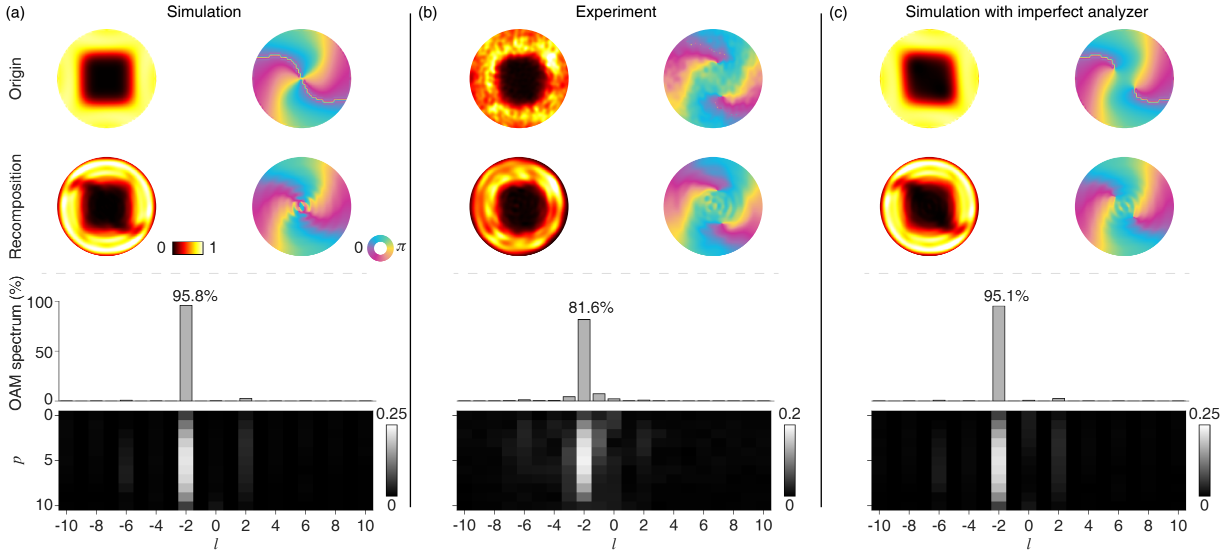

Figure S1. (a) First row: intensity and phase distribution of simulated beam profile. Structural paratmeters are: $h = 337$ nm, $r = 186$ nm and $H = 519$ nm. Second row: recomposed intensity and phase distribution. Third row: histogram for distribution of OAM intensity coefficients. Fourth row: distribution of decomposition coefficient. (b) Experiment results. Phase distribution is measured by off-axis digital hologram reconstruction method. (c) Simulation results with elliptically polarized analyzer ($[1, 0.8i]^T$).

Measured phase distribution of PhC slab is illustrated in the right column of Fig. S1(b). With the measured phase distribution as well as the measured intensity plotted in the second row of Fig. 4(b), LG modal decomposition is performed in experimental data and illustrated in the bottom of Fig. 4(b).

It's shown that $l = -2$ components of experimental data is 81.6%. The composition of $l = -2$ in experimental data is a little bit lower than the theoretical one, which may be caused by measuring error of intensity and phase. Deserve to be mentioned, apart from manufacturing errors, the separation of the measured phase singularity may also be caused by the measuring method and imperfect of optical elements. Since the phase measuring method rely on interference fringes, errors on the measured intensity will introduce fluctuations on extracted phase distribution. While, our generated beam has a doughnut-shaped profile, whose intensity approaches zero around the center, making signal-to-noise greatly fall at the central region. Besides, the imperfect of optical elements will also cause the separation. Before shining on the detector, the generated beam has passed several beam splitters and analyzers. These elements will cause slight error to SOPs of the beam, resulting in the polarization states of incident and the transmission beam non-orthogonal. Unfortunately, we found that the position of central phase singularity is sensitive to the orthogonality of the polarization states of the incident and transmission light. In Fig. S1(c), we changed the ideal circularly polarized analyzer $[1, i]^T$ in simulation into an ellipically polarized one $[1, 0.8i]^T$, making the polarization state of the transmission light slightly deviate orthogonal. It is clearly shown that the central phase singularity obviously separated into two singularities just like that happened in the experiment. Thus, further improvement direction in experiment could focus on how to exactly increase the orthogonality of the incident and transmission light.

[1] M. J. Padgett, Orbital angular momentum 25 years on, Optics express **25**, 11265 (2017).
[2] R. Borghi, F. Gori, and M. Santarsiero, Optimization of laguerre-gauss truncated series, Optics communications **125**, 197 (1996).
[3] Y. Xiao, X. Tang, C. Wan, Y. Qin, H. Peng, C. Hu, and B. Qin, Laguerre-gaussian mode expansion for arbitrary optical fields using a subspace projection method, Optics Letters **44**, 1615 (2019).
[4] N. Verrier and M. Atlan, Off-axis digital hologram reconstruction: some practical considerations, Applied optics **50**, H136 (2011).

II. SUPPORTING FIGURES

An example of above equations is plotted in Fig. S2. In Fig. S2(a), the simulated reflectance spectrum of PhC slab ($a = 460$ nm, $r = 160$ nm, $h = 300$ nm and $H = 360$ nm) at $\boldsymbol{k}_{||}a/2\pi = (0.04, 0)$ is plotted as green circles. Black line is fitting curve with Eq. (8) by tuning fitting parameters γ_0, γ_s and ω_0. Using obtained parameters, cross-polarized conversion efficiency R_c spectrum (green circles) calculated from Eq. (11) as well as simulated R_c (black line) are plotted in Fig. S2(b). From the example, it can be found that both fitting curves and R_c calculated from TCMT theory achieve a good agreement with simulated results. And we can use the measured reflectance spectra to extract the cross-polarized conversion.

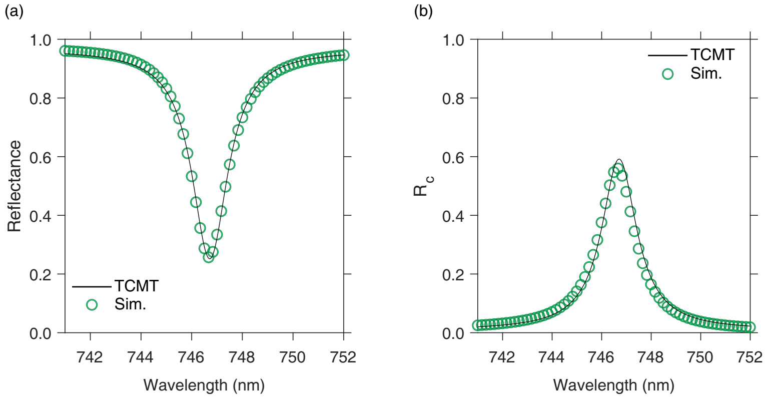

Figure S2. (a) Green circles, the simulated reflectance spectrum of PhC slab at $\boldsymbol{k}_{||}a/2\pi = (0.04, 0)$. Black line, fitting curve with TCMT. (b) Green circles, the simulated cross-polarized conversion efficiency R_c spectrum. Black line, the calculated R_c with TCMT.

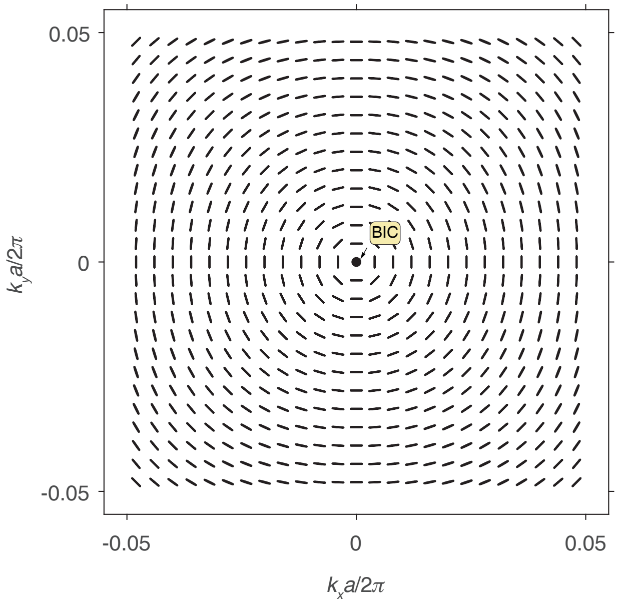

Figure S3. Polarization maps in the vicinity of the Γ point of band TE_2, which has a BIC at center. The charge of the polarization vortex is 1. The proposed band TE_2 is only excited by s-polarized incident light along $\Gamma - X$.

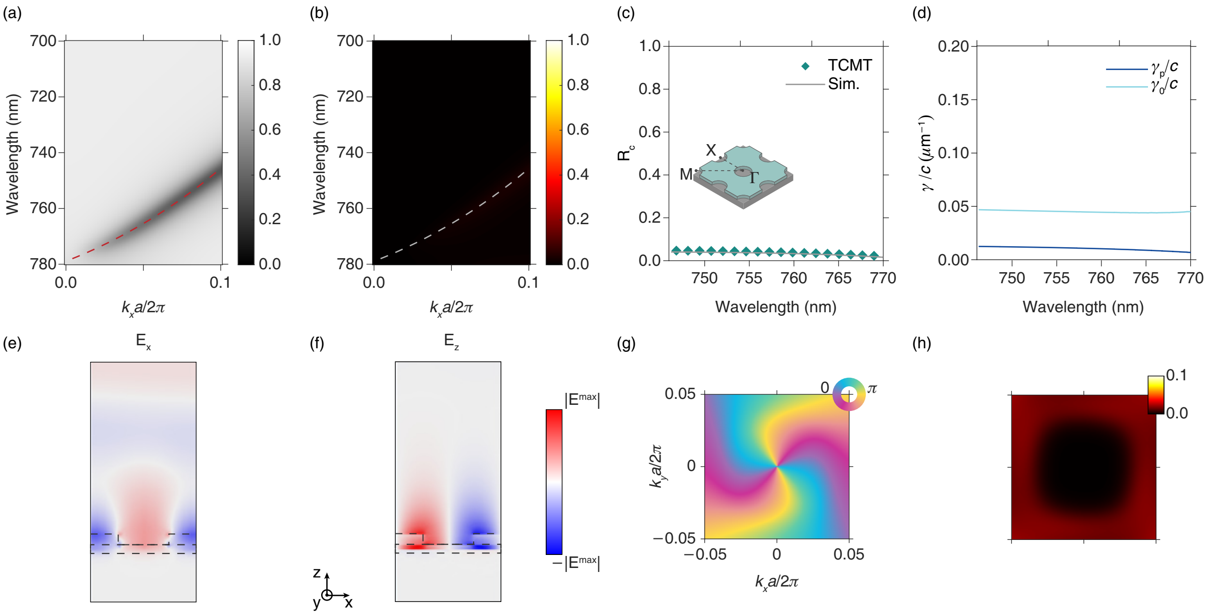

Figure S4. (a) Simulated angle-resolved reflectance spectra of along $\Gamma - X$ under p-polarized incidence of band TM_1. The dashed line points out the band TM_1. The structure parameters: $a = 580$ nm, $r = 140$ nm, $h = 60$ nm and $H = 60$ nm. (b) Simulated angle-resolved cross-polarized conversion efficiency. The efficiency is too small to distinguish at this case. (c) and (d) Gray line stands for simulated cross-polarized conversion efficiency, wathet line and dark blue line for γ_p and γ_0 obtained from TCMT, and green squares for calculated cross-polarized conversion efficiency from Eq. S11. R_c keeps a very low level through the whole band with a much higher absorption loss γ_0 and lower radiation loss γ_p comparing with Fig. 2., whose absorption loss is an order of magnitude larger than our proposed structure. It shows the necessity of mode selection in designing PhC vortex beam generators. (e) and (f) Side view of the E_x and E_z distributions of band TM_1 at $\boldsymbol{k}_{||}a/2\pi = (0.04, 0)$. It can be found that the field gathers around the surface of the silver substrate, causing a large material aborption. (g) and (h) The iso-frequency contour of phase and cross-polarized conversion efficiency at wavelength of 770 nm under circular incidence. The reflected beam is also a vortex beam, while the conversion efficiency now is much lower than 0.1.

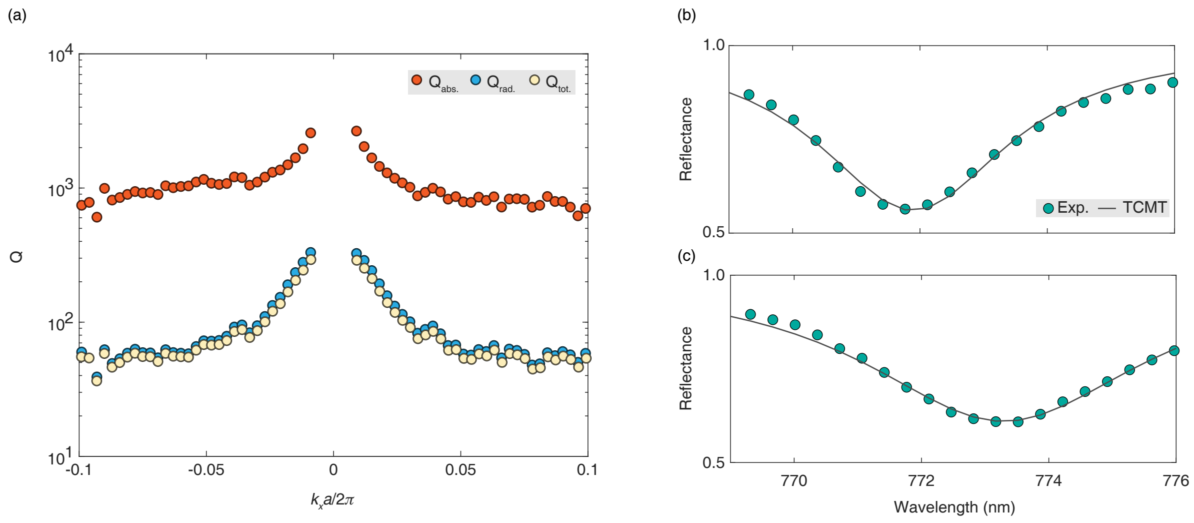

Figure S5. (a) In our work, we use the polarization vortex enabled by BICs to generate optical vortex. For these used resonant modes around BICs, the far-field can excite and the Q value can be extracted from the reflectance spectra by TCMT. In TCMT, the total Q value of a resonator is determined by all loss channels, that is $1/Q_{tot.} = 1/Q_{abs.} + 1/Q_{rad.}+...$, and Q factor of each channel is inversely proportional to its loss in definition, i.e., $1/Q_i = \omega_0/2\gamma_i$, where ω_0 is the resonator frequency. By numerically fitting reflectance spectra with TCMT, obtained Q values in the vicinity of the at-Γ BIC point. Due to the narrow linewidth of the resonance, less Q values near Γ point are extracted. These imperfections will introduce some extra scattering channels beside intrinsic absorption and radiation, which hence reduces the experimental Q factor. (b) and (c) Two examples of the fitted experimental reflectance spectra at $k_x = 0.015 \times 2\pi/a$ and $k_x = 0.02 \times 2\pi/a$.

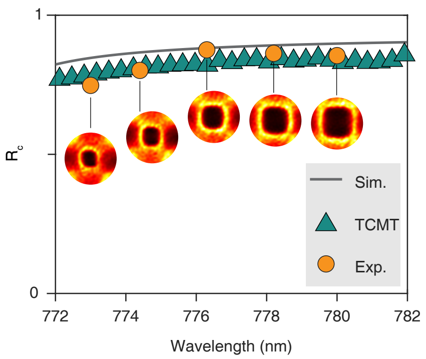

Figure S6. We further measured the cross-polarized conversion efficiency at different wavelengths. The maximal values of conversion efficiency along $\Gamma - X$ direction of every wavelength are extracted and plotted as orange circles. The gray line refers to the simulated on-resonance conversion efficiency, and the green triangles to the maximal conversion efficiency along $\Gamma - X$ obtained by TCMT.

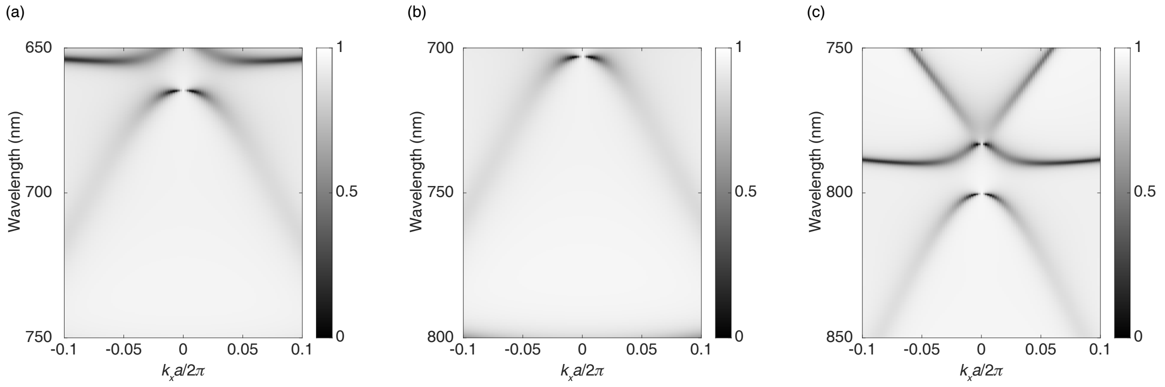

Figure S7. For a 2D photonic crystal slab, the at-Γ BIC point is protected by its rotational symmetry considerations, whose eigenstate is totally decoupled with external channels, having a theoretical infinite lifetime and quality factor. Since this phenomenon is symmetry protected, which means the existence of the BIC point on the second TE-like band discussed in our manuscript doesn't affect by the variation of structural parameters. For PhC slabs with different structural parameters, the at-Γ BIC point following band structures will move to different wavelength region. Here, we illustrated angle-resolved reflectance spectra of PhC slabs with different structural parameters. (a), $a = 395$ nm, $r = 158$ nm, $h = 289$ nm, $H = 445$ nm; (b), $a = 420$ nm, $r = 170$ nm, $h = 305$ nm, $H = 470$ nm; (c), $a = 460$ nm, $r = 170$ nm, $h = 300nm$, $H = 520$ nm.

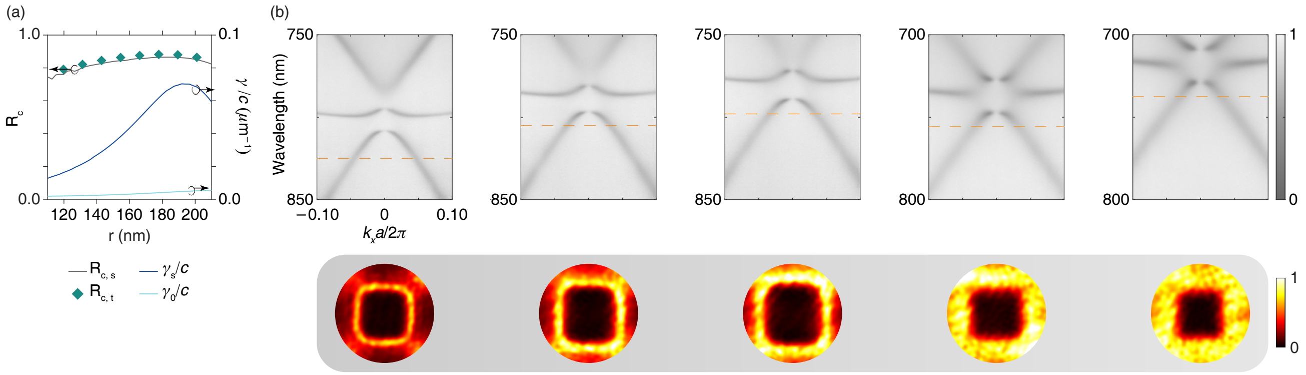

Figure S8. To demonstrate the high efficiency flat step in parameter space, we theoretically designed and experimentally fabricated a series of PhC-slab-type VB generators with different radiuses. Here, thicknesses H of dielectric layers and etched depths h are kept as the constants ($H = 400$ nm, $h = 240$ nm) when radiuses r vary. (a), the on-resonance conversion efficiency Rc at $\mathbf{k}_{||}a/2\pi = (0.04, 0)$, marked as a gray line, keeps a high efficiency as the radius of the hole varying from 110 nm to 210 nm, forming an obvious flat step. Light blue lines and dark blue lines refer to γ_s and γ_0 obtained from TCMT, and green squares to calculated cross-polarized conversion efficiency from TCMT. We chose five sets of parameters on this step ($r = 120$, 135, 145, 190 and 205 nm) to fabricate the sample. (b), the measured angle-resolved spectra of five samples are illustrated with radius increasing from left to right. Note that the wavelength regions of the last two spectra are 700 nm to 800 nm. Below every spectrum, we illustrated an iso-frequency contour of conversion efficiency of every sample. Orange dashed lines on the measured angle-resolved spectra mark the wavelength of the corresponding iso-frequency contours. Illustrated iso-frequency contours of these samples with different radiuses all show a high conversion efficiency, verifying the high efficiency in (a).

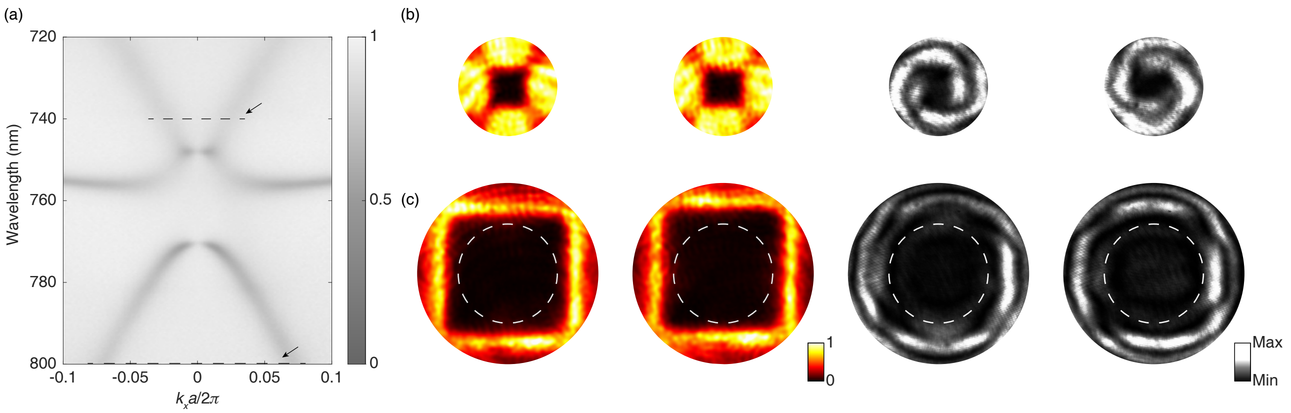

Figure S9. (a), the measured angle-resolved spectrum. (b) and (c), iso-frequency contours and interference fringes at 740.7 nm and 799.8 nm. Measured results with LCP and RCP incidence are plotted in the left and right panel respectively. The black dashed lines in (a) mark two selected wavelengths, whose lengths refer to the k-region illustrated in (b) and (c). The white dashed circle in (c) has the same radius as the patterns in (b). If the wavelength is greatly away from the working bandwidth, the other band will dominate the vortex generation or the beam profile will deviate the desired shape. At 740.7 nm, the upper band with $q = -1$ dominate the vortex generation, whose beam profile becomes cross-shaped and the rotation direction of inference fringes reverses to the ones illustrated in manuscript. At 799.8 nm, radius of the resonance in k-space is out of our working aperture. At this time, the outside part cannot continue to be viewed as the paraxial condition, while the efficiency inside the working aperture is too low to be used. Thus, when the wavelength is clearly above or below the designed wavelength, the spiral wave front will still exist in most of the time, but the beam profile may be out of the shape, making it hard to be used.
